# Effectiveness of mepolizumab in patients with severe eosinophilic asthma: In a real life study

**DOI:** 10.5578/tt.20239918

**Published:** 2023-06-13

**Authors:** İ. Bulut, Z. Yegin Katran, D. Yavuz, A.B. Akyıldız, T. Yakut, C. Örçen, S.S. Mersin

**Affiliations:** 1 Clinic of Allergy Immunology, Süreyyapaşa Chest Diseases-Thoracic Surgery Training and Research Hospital, University of Health Sciences İstanbul, Türkiye; 2 Clinic of Allergy Immunology, Diyarbakır Training and Research Hospital, Diyarbakır, Türkiye; 3 Clinic of Allergy Immunology, Derince Training and Research Hospital, Kocaeli, Türkiye; 4 Clinic of Allergy Immunology, Dr. Ersin Arslan Training and Research Hospital, Gaziantep, Türkiye

**Keywords:** asthma, mepolizumab, real life study

## Abstract

**ABSTRACT:**

Effectiveness of mepolizumab in patients with severe eosinophilic asthma: In a real life study

**Introduction:**

We analyzed the effects of mepolizumab treatment on symptoms, asthma attacks, pulmonary function test parameters peripheral blood eosinophil level, and percentage in patients with severe eosinophilic asthma receiving mepolizumab treatment as the baseline, sixth and twelfthmonth data.

**Materials and Methods:**

The medical records of patients diagnosed with severe eosinophilic asthma and treated with mepolizumab at our clinic were retrospectively reviewed for the period between January 2018 and December 2021. Demographic data of the patients, duration of asthma disease, comorbidities such as a nasal polyp, eosinophilic granulomatous polyangiitis, and nonsteroidal anti-inflammatory drug exacerbated respiratory disease were investigated. A comparison was made of various factors before initiating mepolizumab treatment, as well as at the sixth and twelfth month after treatment initiation. These factors include asthma control test scores, frequency of asthma attacks (including emergency admissions, hospitalizations, and intensive care admissions), peripheral blood eosinophil levels and percentages, and pulmonary function test parameters. Clinic and laboratory parameters that provide a prediction of being a responder and super responder were evaluated.

**Results:**

A total of 21 patients were included in the study. Their mean age was 50.7 ± 11.9 years, and four (19%) were males. The mean duration of asthma diagnosis was 17.5 ±13.7 years. 14 patients (66.7%) were atopic. 4 patients (19%) had nasal polyps and four patients (19%) had NERD. Before mepolizumab, 13 (61.9%) patients had received omalizumab. The duration of receiving mepolizumab treatment was 29.2 ± 9.9 months. A statistically significant dec rease was observed in both the number and percentage of eosinophils at months six and 12 (p< 0.01). There was a statistically significant increase in FEV_1_ values both as a percentage and in milliliters at month 12. There was an increase in both percentage and milliliters in FEF_25-75_ values, but this increase did not reach statistical significance. There was a decrease in service admissions, intensive care admissions, and emergency admissions due to asthma exacerbations. Out of 21 patients, 11 (52.4%) were classified as responders, while 10 (47.6%) were classified as super responders.

**Conclusion:**

Although the number of patients in our study was limited, mepolizumab improved symptom scores in severe eosinophilic asthma, reduced the number of attacks, and improved pulmonary function test values.

## Abstract (tr)

**ÖZ:**

Ağır eozinofilik astımlı hastalarda mepolizumab etkinliği: Bir gerçek yaşam çalışması

**Giriş:**

Ağır eozinofilik astımı olup mepolizumab tedavisi alan hastalarımızda mepolizumab tedavisinin semptomlara, astım ataklarına, solunum fonksiyon testi parametrelerine, periferik kan eozinofil düzey ve yüzdesine etkisini başlangıç, altıncı ay ve 12. ay verileri şeklinde inceledik.

**Materyal ve Metod:**

Ocak 2018-Aralık 2021 tarihleri arasında kliniğimizde mepolizumab ile tedavi edilen ağır eozinofilik astımlı hastaların dosyaları retrospektif olarak incelendi. Hastaların demografik verileri, astım hastalığının süresi, nazal polip, eozinofilik granülomatöz polianjiit ve nonsteroid antienflamatuvar ilaçla alevlenen solunum yolu hastalığı gibi komorbiditeler araştırıldı. Mepolizumab tedavisine başlamadan önce, başladıktan sonraki altıncı ve on ikinci aylarda astım kontrol testi skorları, atak sıklığı (acil başvuru, hastaneye yatış veya yoğun bakım yatışı gerektiren), periferik kan eozinofil düzeyi ve yüzdesi ve solunum fonksiyon testi parametreleri karşılaştırıldı. Yanıtlı ve süper yanıtlı olmanın öngörülmesini sağlayan klinik ve laboratuvar parametreleri değerlendirildi.

**Bulgular:**

Toplam 21 hasta çalışmaya dahil edilmiştir. Hastaların yaş ortalaması 50,7 ± 11,9 yıldı ve dördü (%19) erkekti. Ortalama astım tanı süresi 17,5 ± 13,7 yıldı. Hastaların 14’ü (%66,7) atopikti. Nazal polipli dört hasta (%19) ve NERD’li dört hasta (%19) vardı. Mepolizumab öncesinde 13 (%61,9) hasta omalizumab almıştı. Mepolizumab tedavisi alma süresi 29,2 ± 9,9 aydı. Hem eozinofil sayısında hem de yüzdesinde altıncı ve 12. aylarda istatistiksel olarak anlamlı bir azalma gözlenmiştir (p< 0.01). FEV_1_ değerlerinde hem yüzde hem de mililitre olarak 12. ayda istatistiksel olarak anlamlı bir artış vardı. FEF_25-75_ değerlerinde hem yüzde hem de mililitre olarak artış görülmüş ancak bu artış istatistiksel anlamlılığa ulaşmamıştır. Astım alevlenmeleri nedeniyle servis başvuruları, yoğun bakım başvuruları ve acil başvuruları azalmıştır. Yirmi bir hastanın 11’i (%52,4) yanıtlı olarak sınıflandırılırken, 21 hastanın 10’u (%47,6) süper yanıtlı olarak sınıflandırılmıştır.

**Sonuç:**

Çalışmamızda hasta sayımız az olsa da ağır eozinofilik astımda, mepolizumab semptom skorlarını iyileştirmiş; atak sayılarını azaltmış, solunum fonksiyon testi değerlerinde iyileşme sağlamıştır.

## INTRODUCTION

In cases of difficult asthma, the underlying conditions that contribute to poor control are addressed, and a phenotypic assessment is conducted ( 1 ). Severe eosinophilic asthma (SEA) represents a specific subgroup of asthma characterized by elevated levels of eosinophils in the blood. Patients with SEA need high doses of inhaled corticosteroids plus long-acting β-agonists, and other treatments including oral corticosteroids (OCSs), and anticholinergic or biological agents ( 2 ). Randomized clinical trials (RCTs) have shown that mepolizumab effectively reduces asthma attacks. Additionally, improvements ha ve been observed in the forced expiratory volume in one second (FEV_1_) and the baseline values of the asthma control test (ACT) with the use of mepolizumab ( 2 , 3 ). The objective of this study was to evaluate the impact of mepolizumab on asthma symptom scores, respiratory function test parameters, and blood eosinophil count.

## MATERIALS and METHODS

This retrospective study included 21 SEA patients who were treated with mepolizumab between 2018- 2021. All patients included in the study were monitored for a minimum of one year before initiating mepolizumab treatment. During this period, all patients consistently received high doses of inhaled corticosteroids in combination with long-acting β-agonists, anticholinergic medications, montelukast, and occasionally oral corticosteroids (OCSs). The criteria for starting mepolizumab therapy were evaluated as defined in GINA 2021 guideline. The eligibility criteria for the study were as follows: A) Patients with severe eosinophilic asthma (SEA), B) Patients who had experienced a specific number of severe exacerbations within the last year, and C) Patients with blood eosinophil counts of ≥300 cells/ µL (or ≥150 cells/µL for patients using oral corticosteroids) ( 4 ). The demographic data of the patients, including age, gender, and relevant characteristics such as the duration of asthma disease, presence of comorbidities such as nasal polyps, eosinophilic granulomatous polyangiitis (EGPA), and nonsteroidal anti-inflammatory drug (NSAID)- exacerbated respiratory disease (NERD), were thoroughly investigated and recorded for analysis. Before initiating mepolizumab treatment, various assessments were conducted at both six and 12 months after treatment initiation. These assessments included comparing asthma control test scores, frequency of attacks (including emergency admissions, hospitalizations, and intensive care admissions), peripheral blood eosinophil levels and percentages, as well as pulmonary function test parameters.

### Responder

Patients who demonstrated a 50% or greater reduction in exacerbations necessitating the use of oral corticosteroids (OCSs) for more than three days were considered responders to the treatment at the end of the 12th month.Exacerbations requiring hospitalization, emergency admission, and oral steroid use for at least three days due to asthma were considered clinically significant exacerbations. If patients did not experience any exacerbations requiring the use of oral corticosteroids (OCSs) throughout the 12 months, they were considered super responders to the treatment at the end of the study ( 5 ).

### Responder

Patients who demonstrated a 50% or greater reduction in exacerbations necessitating the use of oral corticosteroids (OCSs) for more than three days were considered responders to the treatment at the end of the 12^th^ month.Exacerbations requiring hospitalization, emergency admission, and oral steroid use for at least three days due to asthma were considered clinically significant exacerbations. If patients did not experience any exacerbations requiring the use of oral corticosteroids (OCSs) throughout the 12 months, they were considered super responders to the treatment at the end of the study ( 5 ).

### Pulmonary Function Test

Spirometry was conducted at the outpatient service of the Allergy and Immunology Clinic at Süreyyapaşa Training and Research Hospital. Forced expiratory volume in 1s (FEV_1_), forced vital capacity (FVC), FEV_1_/FVC ratio and FEF_25-75_ (forced expiratory flow 25-75) were measured as percentages and values. Pulmonary functions tests were performed for all patients following the ATS (American Thoracic Society) recommendations ( 6 ).

### Statistical Analysis

All statistical analyses for the study were conducted using SPSS version 22.0. The differences in means were assessed using the Mann-Whitney U test. Relative risks, odds ratios, and their corresponding 95% confidence intervals were calculated as part of the analysis. For categorical parameters, Chi-square tests and logistic regression analysis were employed. A significance level of p< 0.05 was considered statistically significant in all analyses.

Ethics approval was obtained from the Ethics Committee of the University of Health Sciences, Süreyyapaşa Chest Diseases and Thoracic Surgery Training and Research Hospital. The approval was granted on 08.09.2022, with the protocol code 116.2017.R-252.

## RESULTS

A total of 21 patients were enrolled in the study. Patients who did not complete the one-year follow-up period were excluded from the study. The mean age was 50.7 ± 11.9 years, and four (19%) were males. The mean duration of asthma diagnosis was 17.5 ± 13.7 years. 14 patients (%66.7) were atopic. Four patients (19%) had nasal polyps and four (19%) had NERD. The switching status of patients between biological agents was also examined. Before mepolizumab 13 (61.9%) patients had received omalizumab. The cause of omalizumab discontinuation was treatment failure. The duration of omalizumab treatment was 36.6 ± 25.8 months. The duration of mepolizumab treatment was 29.2 ± 9.9 months ( Table 1 ).

While the eosinophil count was 1217 ± 1509 (150- 6900) before mepolizumab treatment, it was 186 ± 270 (0-1180) at month six and 174 ± 434 (0-2060) at month 12. When the eosinophil values were compared before mepolizumab treatment, and at months six and 12, a statistically significant decrease was observed in both numbers and percentages at months six and 12 (p< 0.01).

In the pulmonary function tests, an increase was observed in the values (milliliters) and percentages of FEV_1_, FVC, FEV_1_/FVC, and FEF_25-75_. There was a statistically significant increase in FEV_1_ values both as a percentage and in milliliters at month 12. When assessing the change in forced vital capacity (FVC), there was a statistically significant increase in milliliters (p< 0.01). However, the percentage increase was not statistically significant at month 12. (p= 0.102) There was an increase in both percentage and milliliters in FEF_25-75_ values, but this increase did not reach statistical significance.

There was a significant increase of 69.2% in asthma control test scores compared to the scores before initiating mepolizumab treatment (p< 0.01). There was a decrease in service admissions, intensive care admissions, and emergency admissions due to asthma exacerbations.

**Table 1:** Demographic and clinical characteristics

Demographic and clinical characteristics	n (%)
Male sex n (%)	4 (19%)
Age, (years, mean ± SD)	50.7 ± 11.9
Mean duration of asthma diagnosis (years, mean ± SD)	17.5 ± 13.7
Atopic n (%)	14 (66.7%)
Nasal polyps n (%)	4 (19%)
NERD n (%)	4 (19%)
Omalizumab before mepolizumab n (%)	13 (61.9%)
Omalizumab treatment duration (months ± SD)	36.6 ± 25.8
Mepolizumab treatment duration (months ± SD)	29.2 ± 9.9

NERD: Nonsteroidal anti-inflammatory drug (NSAID)-exacerbated respiratory disease.

**Table 2:** Comparison of eosinophil count, percentage, pulmonary function test results, and asthma control test (act) scores before mepolizumab treatment, at six months, and at 12 months

	Before mepolizumab (mean ± SD) (min-max)	Month six (mean ± SD) (min-max)	Month 12 (mean ± SD) (min-max)	Month six p	Month 12 p
Blood Eosinophil Count (cell/μL)	1217 ± 1509 (150-6900)	186 ± 270 (0-1180)	174 ± 434 (0-2060)	p< 0.01	p< 0.01
Percentage of Blood Eosinophil	10.7 ± 9.5 (1-44)	2.29 ± 3.1 (0-13)	2.2 ± 5.2 (0-25 )	p< 0.01	p< 0.01
FEV_1_ (mL)	1558 ± 868 (540-3480)	1747 ± 700 (970-3180)	2080 ± 843 (770-3370)	p= 0.16	p= 0.028
FEV_1_(%)	55.6 ± 24.4 (23-99)	63 ± 18 (43-100)	66.2 ± 25.1 (32-104)	p= 0.50	p= 0.028
FVC (mL)	2229 ± 1047 (710-4220)	2559 ± 863 (1460-3930)	2835 ± 949 (1540-4400)	p= 0.01	p< 0.01
FVC (%)	67.5 ± 24.4 (29-111)	76 ± 14 (51-107)	77.8 ± 25.3 (36-118)	p= 0.026	p= 0.102
FEV_1_/FVC	70.6 ± 13.2 (37-92)	68.5 ± 11.5 (45-82)	72.8 ± 25.3 (40-90)	p= 0.894	p= 0657
FEF_25-75_ (mL)	1231 ± 855 (350- 3660)	1356 ± 844 (570-3080)	1652 ± 1104 (380-3630)	p= 0.767	p= 0.156
FEF_25-75_ (%)	33.6 ± 20.7 (11-89)	36 ± 18.3 (17-75)	47 ± 24 (12-88)	p= 0.767	p= 0.156
ACT Score	13.9 ± 3.8 (6-20 )		22.9 ± 1.2 (20-25)		p< 0.01
Service admission with exacerbations	0.9 ± 0.8 (0-3)		0.05 ± 0.2 (0-1)		p< 0.01
ICU admission with exacerbations	1.1 ± 0.3 (0-1)		0		p= 0.157
ED admission with exacerbations	4.9 ± 2.7 (1-11)		1 ± 1.5 (0-7)		p< 0.01

FEV_1_: Forced expiratory volume in 1s; FVC: Forced vital capacity; FEF25-75: Forced expiratory flow 25-75; ACT: Asthma control test; ICU: Intensive care unit; ED: Emergency department; mL: milliliter.

**Table 3:** Responders and super responders

Clinical Characteristics n (%)	Responder ^a^11 (52.4%)	Super Responder 10 (47.6%)
Age, (years mean ± SD)	47.6 ± 14	54.2 ± 8.6
Mean duration of asthma diagnosis (years mean ± SD)	21.6 ± 16.6	13 ± 8.2
Atopic n (%)	11 (100%)	3 (30%)
Nasal polyps n (%)	1 (9%)	3 (30%)
NERD n (%)	1 (9%)	3 (30%)
Omalizumab before mepolizumab n (%)	10 (91%)	3 (30%)
Eosinophil count before mepolizumab (mean ± SD)	1350 ± 1914	1070 ± 971
Percentage of Blood Eosinophil before mepolizumab (mean ± SD)	11.3 ± 12.2	10.1 ± 6.1
FEV_1_ before mepolizumab (mL) (mean ± SD)	1487 ± 875	1539 ± 908
ACT Scores before mepolizumab	14.2 ± 3.3	13.6 ± 4.5

^a^ Responders did not include super responders.

In our group, 11 of 21 (52.4%) patients were classified as responders, and 10 of 21 (47.6%) were classified as super responders. After treatment with mepolizumab, eosinophil count and percentage decreased, pulmonary function test parameters improved, asthma symptom scores increased, and exacerbation rates decreased ( Table 3 ).

## DISCUSSION

In our study, we investigated the impact of mepolizumab on symptom scores, pulmonary function test parameters, and laboratory values in patients with severe eosinophilic asthma. We also analyzed the differences between two groups; responders and super responders. Our analysis revealed that treatment with mepolizumab for six months and one year resulted in significant improvements in all the clinical parameters we assessed. Notably, mepolizumab demonstrated a concurrent improvement in blood eosinophil count and percentage, pulmonary function, asthma exacerbations, and symptom control for all patients.

Improvements were observed in the small airways (FEF_25-75_) and the large airway (FEV_1_) at the end of month six, and month 12 following mepolizumab treatment initiation. The improvement in the large airways reached statistical significance. In numerous studies conducted in our country, it is worth noting that treatment with mepolizumab has consistently led to an increase in FEV_1_ values ( 7 , 8 ). In the MENSA and MUSCA study, it was observed that especially FEV_1_ values showed a statistically significant increase with treatment ( 9 , 10 ). Studies on the effect of mepolizumab on small airways are quite a few. Similar to our study, Sposato et al. also found a significant improvement in FEF_25-75_ percent and milliliters ( 11 ).

Patients with a higher baseline FEV_1_ value before mepolizumab treatment and a shorter duration of asthma were found to be associated with being super-responders. This finding is consistent with the study conducted by Eger et al., who also reported that a higher baseline FEV_1_ and shorter duration of asthma disease were associated with being superresponsive ( 12 ). Before mepolizumab treatment, 13 (61.9%) patients had received omalizumab. In the post hoc analysis of another study that included patients from the MENSA and SIRIUS studies with mepolizumab, it was observed that mepolizumab, regardless of the previous use of omalizumab, reduced the use of oral corticosteroids (OCSs). In both the MENSA and SIRIUS studies, significant improvement in quality of life measures was observed with mepolizumab treatment, regardless of previous use of omalizumab ( 10 , 13 , 14 ). In the OSMO study, patients who had previously been receiving omalizumab treatment but switched to mepolizumab due to inadequate clinical response showed clinically significant improvements in asthma control and exacerbation rate. These findings align with the results of our study, demonstrating the effectiveness of mepolizumab in managing uncontrolled severe eosinophilic asthma ( 15 ). In the study conducted by Yılmaz et al. in our country, it was reported that there were no significant changes in FEV_1_ and FEF_25-75_ values at 12-24 and 52 weeks with mepolizumab treatment ( 16 ). However, it is worth noting that despite the reduction in oral corticosteroid dose, the FEV_1_ and FEF_25-75_ values did not decrease.

In eosinophilic airway inflammation, T helper two lymphocytes and innate lymphocyte type 2 are predominant ( 17 ). These cells are responsible for interleukin (IL) 4, IL-5, IL-9, and IL-13 as well as abnormal immunoglobulin production, i.e., atopy, eosinophilia, and mast cell enlargement. In other words, allergic and eosinophilic phenotypes often coexist and overlap ( 18 ). Fourteen (66.7%) patients included in the study were atopic; 11 (78.5%) of these patients had previously received omalizumab treatment and switched to mepolizumab when no clinical response was obtained. In the post hoc analysis of the MENSA study, 52.7% of patients receiving mepolizumab treatment were atopic. However, this atopy status was not associated with clinical response to mepolizumab ( 19 ).

In real life, an increase of three points or more in the Asthma Control Test (ACT) is considered clinically significant ( 20 ). In our study, treatment with mepolizumab resulted in a significant improvement in ACT scores at the end of one year. (13.9-22.9; p< 0.05) In the study conducted by Spasoto et al., it was observed that treatment with mepolizumab resulted in a statistically significant increase of at least five points in ACT scores ( 21 ).

Different adverse drug reactions associated with mepolizumab have been reported in the literature ( 22 ). However, it is important to note that our study was retrospective, and therefore, our data on adverse reactions are limited. In one patient, symptoms such as dizziness, lightheadedness, and chest pain were noted within the first 10 minutes after mepolizumab treatment. During the monitoring of the patients, no significant changes were observed in serum troponin levels, electrocardiographic measurements, and blood pressure. Tryptase levels were found to be within the normal range. For the subsequent visits, prick tests and intradermal tests were performed using mepolizumab, and the results were negative, indicating no allergic reaction. Consequently, the drug was administered with desensitization protocols during each administration to ensure the safety of the patient. Currently, mepolizumab is administered with a desensitization protocol. Another patient in our study developed a maculopapular drug eruption after receiving mepolizumab treatment. However, this patient had concurrent use of other drugs. The prick test, intradermal test, and patch test with mepolizumab were negative. The drug was given with desensitization at the next visit. There was no recurrence of symptoms in either patient. However, as the prick test, intradermal test, and patch test all yielded negative results, it was not possible to conclude that the current conditions were directly related to the drug. Furthermore, no side effects necessitating the discontinuation of the drug were observed in any of the patients’ files. In a study conducted in our country, a drug discontinuation rate of 2.4% was observed, but it could not be definitively established that the side effects were directly attributed to mepolizumab ( 23 ). It is worth noting that the safety profile of mepolizumab is generally favorable ( 24 ). Although our patient sample size is small and our data on minor side effects are limited, we have successfully continued treatment in all of our patients.

Considering the limitations of the study, which was retrospective and had a relatively small number of patients, it is important to note that when we grouped them into responders and super responders to treatment, the sample size became even more limited. While the results we obtained are encouraging, particularly for the super responders, it is necessary to analyze larger patient cohorts in order to draw more robust conclusions.

In this real-life study, mepolizumab improved symptom scores, pulmonary function tests, and laboratory values. At the end of one year, a clinically significant improvement was observed in the number and percentage of blood eosinophils, FEV1 in milliliters and percentage, FVC in milliliters, ACT score, and the frequency of attacks requiring hospitalization and emergency admission.

## Acknowledgments

I would like to express my gratitude and appreciation to all of my colleagues with whom I have had the privilege of working together.

## Ethical Committee Approval

This study was approved by University of Health Sciences Süreyyapaşa Chest Diseases-Thoracic Surgery Training and Research Hospital Non-Invasive Clinical Ethics Committee. (Decision no: 116.2007.R-251, Decision date: 08.09.2022).

## CONFLICT of INTEREST

The authors declare that they have no conflict of interest.

## AUTHORSHIP CONTRIBUTIONS

Concept/Design: All of authors

Analysis/Interpretation: All of authors

Data acqusition: All of authors

Writing: All of authors

Clinical Revision: All of authors

Final Approval: All of authors

## References

[bb0005] Agache I., Beltran J., Akdis C., Akdis M., Canelo-Aybar C., Canonica G.W. (2020). Efficacy and safety of treatment with
biologicals (benralizumab, dupilumab, mepolizumab,
omalizumab and reslizumab) for severe eosinophilic asthma. A systematic review for the EAACI
Guidelines - recommendations on the use of biologicals in severe asthma.

[bb0010] Li H., Zhang Q., Wang J., Gao S., Li C., Wang J. (2021). Realworld effectiveness of mepolizumab in severe eosinophilic
asthma: A systematic review and meta-analysis..

[bb0015] Cabon Y., Molinari N., Marin G., Vachier I., Gamez A.S., Chanez P. (2017). Comparison of anti-interleukin-5 therapies
in patients with severe asthma: Global and indirect
meta-analyses of randomized placebo-controlled trials.

[bb0020] Reddel H.K., Bacharier L.B., Bateman E.D., Brightling C.E., Brusselle G.G., Buhl R. (2021). Global initiative for asthma
strategy 2021: Executive summary and rationale for key
changes.

[bb0025] Kavanagh J.E., d’Ancona G., Elstad M., Green L., Fernandes M., Thomson L. (2020). Real-world effectiveness and the
characteristics of a “super-responder” to mepolizumab in
severe eosinophilic asthma.

[bb0030] Graham B.L., Steenbruggen I., Miller M.R., Barjaktarevic I.Z., Cooper B.G., Hall G.L. (2019). Standardization of spirometry
2019 update. An official American Thoracic Society and
European Respiratory Society Technical Statement.

[bb0035] Özdel Öztürk B., Yavuz Z., Eraslan D., Mungan D., Demirel Y.S., Aydın Ö., Sin B.A., Bavbek S. (2022). Mepolizumab is an effective option in severe eosinophilic asthma regardless of
baseline features: Single-center real-life data.

[bb0040] Cakmak M.E., Öztop N., Yeğit O.O., Özdedeoğlu Ö. (2023). Evaluation of the clinical features and laboratory data of
patients with severe eosinophilic asthma classified as
super-responders, partial responders, or nonresponders to
mepolizumab treatment: A real-life study.

[bb0045] Chupp G.L., Bradford E.S., Albers F.C., Bratton D.J., Wang-Jairaj J., Nelsen L.M. (2017). Efficacy of mepolizumab add-on therapy on health-related quality of life and markers of asthma
control in severe eosinophilic asthma (MUSCA): A randomised, double-blind, placebo-controlled, parallel-group,
multicentre, phase 3b trial.

[bb0050] Ortega H.G., Liu M.C., Pavord I.D., Brusselle G.G., FitzGerald J.M., Chetta A. (2014). Mepolizumab treatment in patients with severe eosinophilic asthma.

[bb0055] Sposato B., Camiciottoli G., Bacci E., Scalese M., Carpagnano G.E., Pelaia C. (2020). Mepolizumab effectiveness on small airway obstruction, corticosteroid sparing and maintenance therapy step-down in real life.

[bb0060] Eger K., Kroes J.A., Ten Brinke A., Bel E.H. (2021). Long-term therapy response to anti-IL-5 biologics in severe asthma-a real-life evaluation.

[bb0065] Bagnasco D., Milanese M., Rolla G., Lombardi C., Bucca C., Heffler E. (2018). The North-Western Italian experience with anti IL-5 therapy amd comparison with regulatory trials.

[bb0070] Magnan A., Bourdin A., Prazma C.M., Albers F.C., Price R.G., Yancey S.W., Ortega H. (2016). Treatment response with mepolizumab in severe eosinophilic asthma patients with previous omalizumab treatment..

[bb0075] Chapman K.R., Albers F.C., Chipps B., Muñoz X., Devouassoux G., Bergna M. (2019). The clinical benefit of mepolizumab replacing omalizumab in uncontrolled severe eosinophilic asthma.

[bb0080] Yılmaz İ., Nazik Bahçecioğlu S., Türk M., Tutar N., Paçacı Çetin G., Arslan B. (2021). Effectiveness of mepolizumab therapy on symptoms, asthma exacerbations, steroid dependence,
and small airways in patients with severe eosinophilic asthma.

[bb0085] Heffler E., Blasi F., Latorre M., Menzella F., Paggiaro P., Pelaia G. (2019). The Severe Asthma Network in Italy: Findings and perspectives.

[bb0090] Lambrecht B.N., Hammad H., Fahy J.V. (2019). The cytokines of asthma.

[bb0095] Prazma C.M., Idzko M., Douglass J.A., Bourdin A., Mallett S., Albers F.C. (2021). Response to mepolizumab treatment in
patients with severe eosinophilic asthma and atopic phenotypes.

[bb0100] Schatz M., Kosinski M., Yarlas A.S., Hanlon J., Watson M.E., Jhingran P. (2009). The minimally important difference of the asthma control test.

[bb0105] Sposato B., Scalese M., Camiciottoli G., Carpagnano G.E., Pelaia C., Santus P. (2021). Mepolizumab effectiveness and allergic status in real life.

[bb0110] Wenzel S. (2023). Treatment of severe asthma in adolescents and adults.

[bb0115] Bahçecioğlu S.N., Türk M., Atayık E., Çetin G.P., Arslan B., Gülmez İ. (2022). Mepolizumab-related adverse events in severe eosinophilic asthma: A real-life study.

[bb0120] Çetin G.P., Arslan B., Yılmaz İ. (2022). Safety profiles of biological therapies used in asthma treatment.

